# Betulinic Acid Prevents the Acquisition of Ciprofloxacin-Mediated Mutagenesis in *Staphylococcus aureus*

**DOI:** 10.3390/molecules24091757

**Published:** 2019-05-07

**Authors:** Alexsander Rodrigues Carvalho Junior, Arthur Lima de Berredo Martins, Brenda da Silva Cutrim, Deivid Martins Santos, Hermerson Sousa Maia, Mari Silma Maia da Silva, Adrielle Zagmignan, Maria Raimunda Chagas Silva, Cristina de Andrade Monteiro, Giselle Maria Skelding Pinheiro Guilhon, Antônio José Cantanhede Filho, Luís Cláudio Nascimento da Silva

**Affiliations:** 1 Programa de Pós-graduação, Universidade Ceuma, São Luís, Maranhão 65075-120, Brazil; arcarvalhojr@gmail.com (A.R.C.J.); arthurlbm@hotmail.com (A.L.d.B.M.); bsilvadc@gmail.com (B.d.S.C.); deivid.martinss98@gmail.com (D.M.S.); maiahermerson@gmail.com (H.S.M.); msmaia@live.com (M.S.M.d.S.); adriellyzagmignan@hotmail.com (A.Z.); marirah@gmail.com (M.R.C.S.); cristina.monteiro@ceuma.br (C.d.A.M.); 2 Instituto de Ciências Exatas e Naturais, Universidade Federal do Pará, Bélem 66075-110, Pará, Brazil; giselle@ufpa.br; 3 Departamento de Química, Instituto Federal do Maranhão, São Luís, Maranhão 65030-005, Brazil; prof.antoniofilho@ifma.edu.br

**Keywords:** natural products, drug helpers, quinolones, DNA damage, drug resistance

## Abstract

The occurrence of damage on bacterial DNA (mediated by antibiotics, for example) is intimately associated with the activation of the SOS system. This pathway is related to the development of mutations that might result in the acquisition and spread of resistance and virulence factors. The inhibition of the SOS response has been highlighted as an emerging resource, in order to reduce the emergence of drug resistance and tolerance. Herein, we evaluated the ability of betulinic acid (BA), a plant-derived triterpenoid, to reduce the activation of the SOS response and its associated phenotypic alterations, induced by ciprofloxacin in *Staphylococcus aureus*. BA did not show antimicrobial activity against *S. aureus* (MIC > 5000 µg/mL), however, it (at 100 and 200 µg/mL) was able to reduce the expression of *recA* induced by ciprofloxacin. This effect was accompanied by an enhancement of the ciprofloxacin antimicrobial action and reduction of *S. aureus* cell volume (as seen by flow cytometry and fluorescence microscopy). BA could also increase the hyperpolarization of the *S. aureus* membrane, related to the ciprofloxacin action. Furthermore, BA inhibited the progress of tolerance and the mutagenesis induced by this drug. Taken together, these findings indicate that the betulinic acid is a promising lead molecule in the development helper drugs. These compounds may be able to reduce the *S. aureus* mutagenicity associated with antibiotic therapies.

## 1. Introduction

The misuse of drugs usually prescribed for treatment of infectious diseases has been crucial for the development of bacterial resistance to antibiotics [1,2]. This phenomenon leads to faster propagation of multidrug resistance bacteria and constitutes one of the greatest challenges to public health, worldwide [3]. In addition, several evidences have indicated that bacteria, such as *Staphylococcus aureus*, have acquired several mechanisms to ensure their survival in adverse conditions, leading to drug tolerance and persistence [4,5,6]. 

In general, the classic concept indicates that bacterial resistance and virulence factors arise from a pre-existing selection of mutants in a bacterial population treated with antibiotics [2,7]. However, recent studies have shown the emergence of de novo mutations, after the exposure of bacteria in non-lethal stress conditions [8]. This event is known as “adaptative resistance” [9,10], and it is related to the triggering of the SOS system, which leads to increased rates of recombination and mutation, affecting the evolution and dissemination of bacterial resistance [11,12]. This event is known as “adaptative resistance” [9,10], and it is related to the triggering of the SOS system, which leads to increased rates of recombination and mutation, affecting the evolution and dissemination of bacterial resistance [11,12].

The SOS response consists of an orchestrated pathway, performed by a multiprotein complex that is coordinately activated by the bacteria, in response to various conditions that induce DNA damage or blockage, in the cell replication (as antibiotic treatment) [13,14,15]. This pathway is activated by the accumulation of single-stranded DNAs (ss-DNA) that are bound by RecA, and this complex induces the auto-cleavage of the LexA protein [16,17]. After this, the expression of SOS-related genes is activated, resulting in the inhibition of the cell division process, in order to repair the DNA [13,18]. However, when the integrity of both DNA strands is affected, the expression of error-prone DNA polymerases is activated and their low fidelity might result in bacterial mutagenesis [19,20,21].

The activation of SOS response in *S. aureus* by drugs (such as quinolones and Mitomycin C) and hydrogen peroxide, has been shown to increase the frequency of small colony variants (SCVs), a sub-population of slow-growing cells that are currently associated with chronic and recurrent infections, which are extremely tolerant to antibiotics and can persist into the host cells [22,23]. SOS response has been also associated with the release of extracellular membrane vesicles from *S. aureus* lysogenic strains, these structures contribute to bacterial virulence and drug resistance [24]. Recently, it has been demonstrated that some drugs used for cancer therapy can also enhance the emergence of resistant strains, through the induction of the SOS pathway [25]. This panorama suggests that SOS-related proteins are interesting targets for the development of drug helpers, and it has encouraged the search of compounds able to inhibit this pathway [26,27,28].

**Figure 1:**
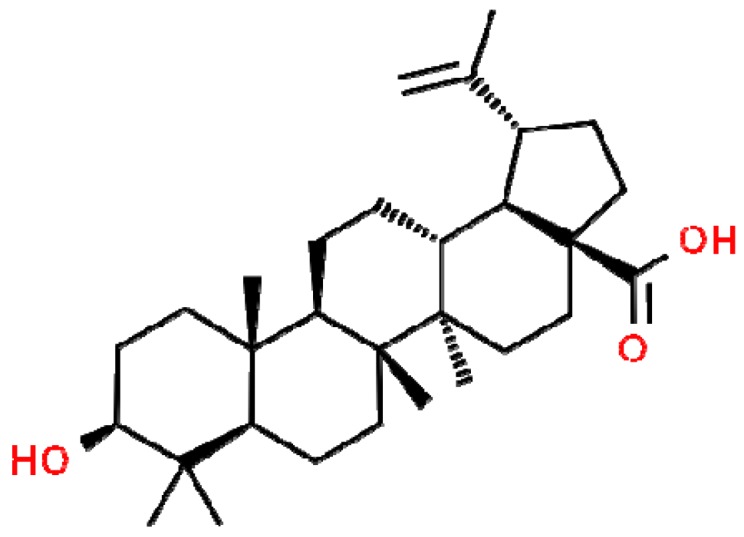
Chemical structure of betulinic acid. This structure was obtained from Chemspider (http://www.chemspider.com; ChemSpider ID58496).

Natural products are recognized as source of lead compounds for the pharmaceutical industry; an example of its active molecule is betulinic acid (BA), a pentacyclic lupane-type triterpenoid found in some plants (Figure 1). This compound is reported to be an antiviral agent and have antidiabetic, antitumoral, antihyperlipidemic, and anti-inflammatory activities [29,30,31,32,33,34]; however, the reports about its antimicrobial activity are controversial [35,36,37,38]. Taking into account the medicinal properties of BA, we evaluated its effects on the SOS response induced by ciprofloxacin, and evaluated whether this action was associated to a reduction of the progress of drug-tolerance in *S. aureus*. BA used in this study was extracted from the leaves of *Eugenia flavescens* DC [39].

## 2. Results

### 2.1. BA Inhibits Ciprofloxacin-Mediated SOS Response

Prior to the evaluation of the effects of BA on ciprofloxacin-mediated SOS response, we evaluated its antimicrobial activity by determining the Minimal Inhibitory Concentration (MIC). BA did not show any antimicrobial action against the tested *S. aureus* strains (MIC > 5000 µg/mL). While the MIC values for ciprofloxacin were 0.078 µg/mL and 0.0195 μg/mL, against the *S. aureus* strains ATCC 6538 and 432170, respectively (Table 1). In addition, BA did not inhibit the growth of other microorganisms, such as *Candida albicans*, *Cryptococcus gattii*, and *Pseudomonas aeruginosa* (data not shown).

**Figure 2:**
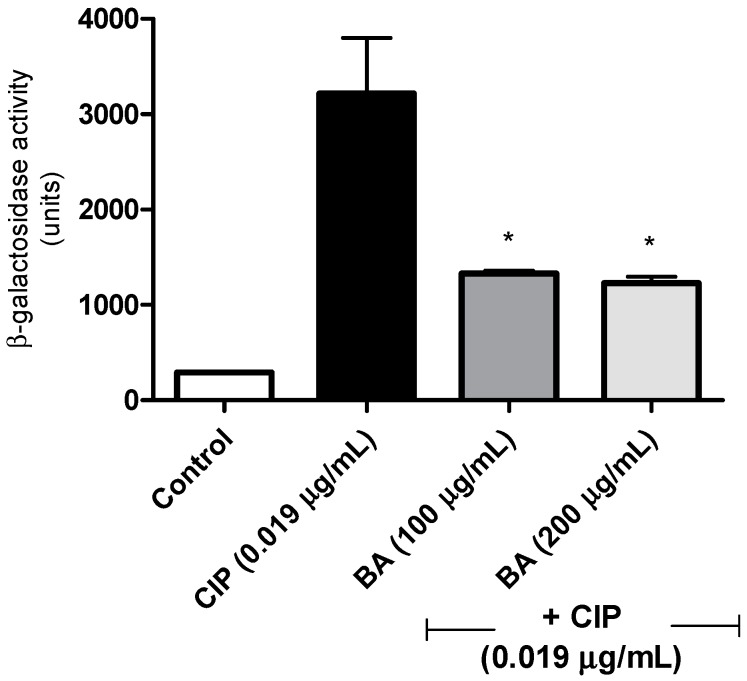
Effect of betulinic acid in the expression of *S. aureus recA* induced by ciprofloxacin. The strain was incubated with ciprofloxacin (0.019 µg/mL) alone or in combination with betulinic acid (100 or 200 µg/mL). The expression of *recA* were measured after 3 h, using a derivative *S. aureus* 8325-4 strain carrying a *recA::lacZ* fusion. β-galactosidase activity was measured using 2-Nitrophenyl β-d-galactopyranoside (ONPG). CIP—Ciprofloxacin; BA—betulinic acid. * indicates statistical differences related to the ciprofloxacin-treated cells (*p* < 0.05).

The SOS inhibition assay was based on the *recA* expression, using the strain *S. aureus* 8325-4 *recA::lacZ* [40]. As expected, after 3 h, the *S. aureus* cells grown in the presence of the sub-inhibitory concentration (sub-MIC) of ciprofloxacin, showed a high expression of *recA*, when compared with the non-treated cells (*p* < 0.05), indicating the activation of the SOS pathway. On the other hand, the co-treatment of this strain with BA and ciprofloxacin, resulted in a significant reduction (almost 60% for BA at 200 µg/mL or 100 µg/mL) in the *recA* expression, when compared to the ciprofloxacin-treated cells (*p* < 0.05) (Figure 2). BA itself did not affect the expression of *recA* gene, in relation to the control cells. In our screening we also evaluated the effects of lupeol, another pentacyclic lupane-type triterpenoid isolated from *E. flavescens* [39], however, it did not affect the expression of *rec A* induced by ciprofloxacin. Lupeol also did not reduce the growth of *S. aureus* (data not shown).

**Figure 3:**
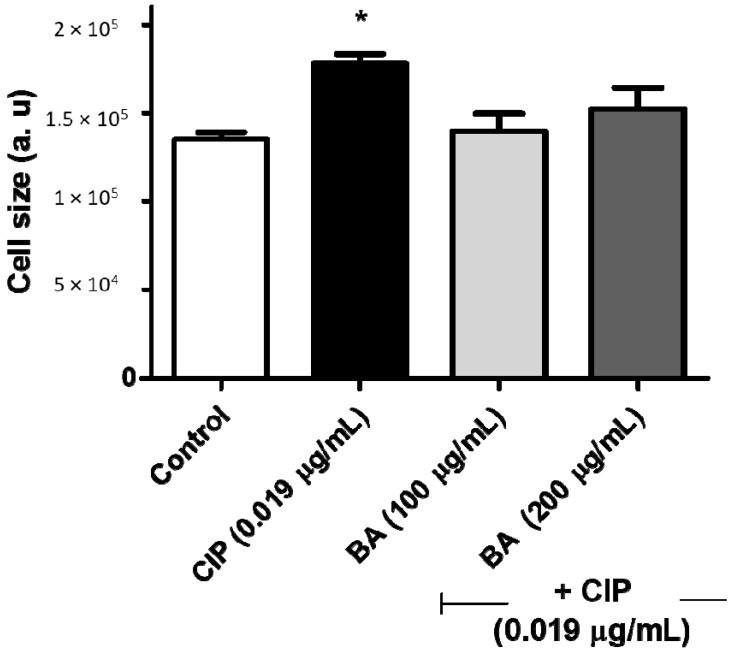
Effects of betulinic acid in the increase of cell volume induced by ciprofloxacin. *S. aureus* ATCC 6538 was incubated with ciprofloxacin (0.019 µg/mL) alone or in combination with betulinic acid (100 or 200 µg/mL) and after 3 h, the cell volume was determined using flow cytometry. CIP—ciprofloxacin; BA—betulinic acid. * indicates statistical differences related to the untreated cells (*p* < 0.05).

**Figure 4:**
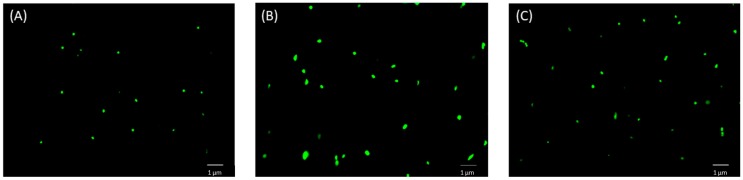
Effect of betulinic acid in the cell volume of ciprofloxacin treated *S. aureus* measured by fluorescence microscopy. *S. aureus* ATCC 6538 was incubated with ciprofloxacin (0.019 µg/mL) alone, or in combination with betulinic acid (200 µg/mL). After 3 h, the cells were labeled with acridine orange and analyzed using fluorescence microscopy. (**A**) Non-treated cells; (**B**) *S. aureus* treated with ciprofloxacin (0.019 µg/mL); (**C**) *S. aureus* treated with ciprofloxacin (0.019 µg/mL) and betulinic acid (200 µg/mL).

To provide more insights into the inhibitory action of BA towards SOS response induced by ciprofloxacin, we evaluated whether the co-treatment with these agents could affect the cell size of *S. aureus*. As earlier explained, during the SOS response, there is a blockage on the cell cycle, in an attempt to repair DNA damage, resulting in an increase of the cell size [41]. Therefore, cells incubated with ciprofloxacin showed an increased cell size, when compared to the non-treated cells (Figure 3). The co-treatment with BA significantly reduced this effect (around 20% for both concentrations). These findings were also confirmed by the fluorescence microscopy (Figure 4).

### 2.2. BA Enhances the Activity of Ciprofloxacin Against S. aureus

The suppression of SOS pathway has been associated with the potentialization of antibiotic actions and reversal of drug resistance [26,42]. Thus, we evaluated whether the inhibitory action of BA on *recA* expression mediated by ciprofloxacin, could increase the susceptibility of *S. aureus* to this drug. Our results showed that BA enhanced the ciprofloxacin action against both strains. The co-treatment with BA reduced the MIC values of ciprofloxacin by half (100 μg/mL) and one-quarter (200 μg/mL) (Table 1).

### 2.3. The Effect of BA on Bacterial Cell Membrane Potential

**Figure 5:**
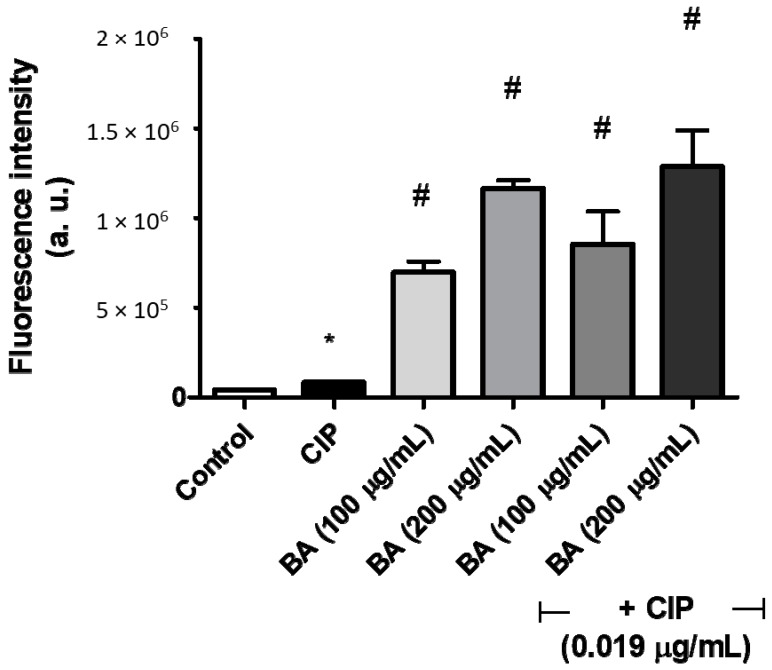
Effect of betulinic acid alone or in combination with ciprofloxacin on the bacterial membrane potential. *S. aureus* ATCC 6538 was incubated with ciprofloxacin (0.019 µg/mL) alone or in combination with betulinic acid (100 or 200 µg/mL). After 3 h, the cells were labeled with Rhodamine 123 and analyzed using fluorescence microscopy. CIP—ciprofloxacin; BA—betulinic acid. * indicates statistical differences related to the untreated cells (*p* < 0.05). ^#^ indicates statistic differences related to the ciprofloxacin-treated cells (*p* < 0.05).

The effects of BA (with or without ciprofloxacin) on cell membrane, were evaluated using the fluorescent dye Rhodamine123 (Figure 5). The ciprofloxacin treatment caused hyperpolarization on the *S. aureus* cell membrane (variation index (VI) of 1.05). Interestingly, the cells treated only with BA exhibited even higher levels of hyperpolarization (VI indices of 26.43 and 16.10 for BA at 200 μg/mL and 100 μg/mL, respectively) than those treated with ciprofloxacin alone. In addition, the bacteria co-treatment with BA and CIP also showed membrane hyperpolarization (VI indices: 28.33 and 20.64, for co-treatment with BA at 200 μg/mL and 100 μg/mL, respectively).

### 2.4. BA Affects the SOS-Mediated Mutagenesis Promoted by Ciprofloxacin

**Figure 6:**
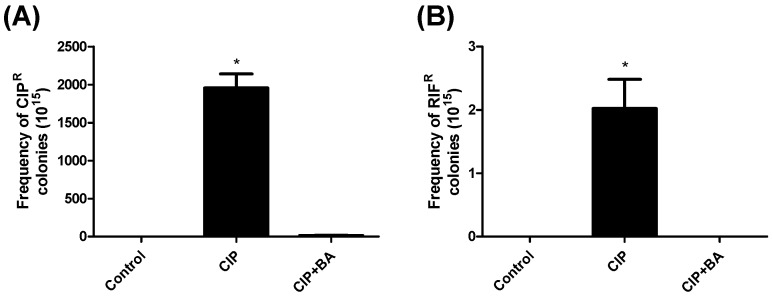
Effect of betulinic acid (200 µg/mL) in the reduction of mutation frequency induced by ciprofloxacin. *S. aureus* ATCC 6538 was incubated with ciprofloxacin (0.019 µg/mL) alone or in combination with betulinic acid (200 µg/mL). After 48 h, the mutants inside the bacterial population were selected using MH (Mueller–Hinton) agar, supplemented with ciprofloxacin or rifampicin. (**A**) Frequency of ciprofloxacin-resistant colonies (CIP^R^) induced by ciprofloxacin; (**B**) Frequency of rifampicin-resistant (RIF^R^) colonies induced by ciprofloxacin. BA—betulinic acid; CIP—ciprofloxacin; RIF—rifampicin. * indicates statistical differences related to the untreated cells (*p* < 0.05).

Sub-MIC values of the drugs are recognized to induce bacterial mutagenesis and tolerance in the pathways related to the SOS response [43]. We determined whether the suppression of *recA* expression mediated by BA, could affect the frequency of ciprofloxacin-induced mutagenesis. The mutants inside the bacterial population were selected using MH (Mueller–Hinton) agar, supplemented with ciprofloxacin or rifampicin. The treatment with sub-MIC concentrations of ciprofloxacin resulted in increased appearance of both ciprofloxacin-resistant (CIP^R^) and rifampicin-resistant (RIF^R^) colonies, compared to the untreated cells. However, the co-treatment with ciprofloxacin and BA (200 μg/mL) significantly decreased the mutation frequency for both drugs, to levels similar to those observed for the untreated cells (Figure 6).

### 2.5. BA Reduces the Profile of Drug Tolerance Caused by Ciprofloxacin

**Figure 7:**
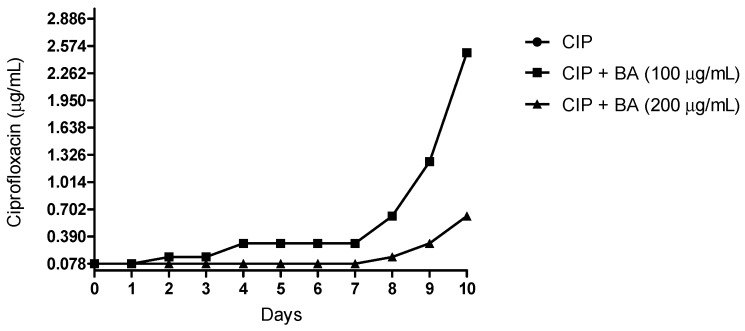
Effects of betulinic acid on the drug tolerance induced by ciprofloxacin. CIP—ciprofloxacin; BA—betulinic acid. *S. aureus* ATCC 6538 was successively grown in the presence of sub-minimal inhibitory concentration (MICs) of ciprofloxacin and BA (200 μg/mL or 100 μg/mL) and after each cycle, the MIC for ciprofloxacin was determined. CIP—ciprofloxacin; BA—betulinic acid.

The ability of BA to alter the profile of tolerance acquisition towards ciprofloxacin was also measured. First, all groups were treated with sub-inhibitory concentrations of ciprofloxacin (MIC/2). After two passages, the group treated only with ciprofloxacin already increased the MIC values. On the fourth day of treatment, the same group showed an MIC of 0.312 μg/mL (4-folds increase), while the cells co-treated with BA (200 μg/mL) and ciprofloxacin did not change the MIC values (0.078 μg/mL). On the eighth day, the MIC of the ciprofloxacin-treated cells changed to 0.624 μg/mL (8-folds) and the MIC for the group co-treated with BA (200 μg/mL) and ciprofloxacin, exhibited a two-folds increase (0.156 μg/mL). At the end of the experiment, the MIC for ciprofloxacin-treated *S. aureus* was 32-folds higher (2.496 μg/mL). The group treated with BA (200 μg/mL) and ciprofloxacin had 8-folds increase in MIC on the tenth day (0.624 μg/mL) (Figure 7). When tested at 100 μg/mL, BA did not inhibit the development of tolerance induced by ciprofloxacin.

## 3. Discussion

The worldwide spread of multidrug resistant bacteria has limited the effectiveness of antimicrobial therapy, leading to a cycle where the overuse of antibiotics leads to the emergence of new multidrug-resistant bacteria (by selection of pre-existing mutants or induction of de novo mutation) that demand the use of higher doses [13,44]. SOS-mediated mutagenicity has been reported for drugs that directly target bacterial DNA structure and replication (such as ciprofloxacin, mitomycin C, and other used in cancer treatment), but also for other class of antimicrobials, such as aminoglycosides and beta-lactams [22,25,45,46]. However, despite this important role played by the SOS system in the adaptation and acquisition of bacterial resistance to antimicrobials, only a handful of studies have focused on the prospect of compounds able to inhibit this pathway in *S. aureus*. Herein, we report that betulinic acid suppressed the effects of ciprofloxacin-mediated SOS activation and its consequences on drug tolerance and mutagenicity. 

Initially, we evaluated the antimicrobial action of BA, however, this compound did not affect the growth of tested *S. aureus* strains, *P. aeruginosa*, *C. gattii*, and *C. albicans*. The literature presents controversial data about the antimicrobial action of BA. For example, some authors sustain that BA is not active against *Bacillus subtilis*, *C. albicans*, *Escherichia coli*, and *S. aureus* [35], corroborating with our findings. On the other hand, other studies have reported a low activity (MIC ≥ 128 μg/mL) of BA against *Bacillus cereus, Enterococcus fecalis*, *E. coli*, *Listeria monocytogenes, Pseudomonas aeruginosa*, *Salmonella enterica*, and *S. aureus* [47,48,49,50]. Contrasting results were reported by Chung et al. (2014), who found MIC values ranging from 4 to 64 μg/mL against different *S. aureus* strains; this action was associated with multiple targets, such as ABC transporters, two-component systems, and ribosomal assembly [36].

We also investigated the application of BA as a drug helper, by analyzing its effects of *recA* expression induced by ciprofloxacin. RecA is the first protein on the SOS cascade and it has been pointed to be an efficient target to improve the efficacy of the marketed antimicrobials [26,42,51]. Our results demonstrated that BA reduced the levels of *recA* transcription induced by ciprofloxacin. Interestingly, lupeol a compound structurally related to BA (both are pentacyclic lupane-type triterpenoids) was not able to reduce *recA* expression. The effects of BA on *recA* expression was linked to an increase (2 or 4 folds) in the susceptibility of *S. aureus* for ciprofloxacin. Similar effects were reported for two phthalocyanine tetrasulfonate-based compounds, which, by targeting RecA were able to increase the activity of ciprofloxacin (and other drugs) towards *S. aureus*, *E. coli*, *P. aeruginosa*, and *Enterococcus faecalis* [52]. 

An important event of the SOS cascade is the increase of cell size was due the inhibition of cell division, in order to ensure DNA repair [53]; and several works have shown that the development of filamentation in *E. coli* are related with this pathway. In this sense, cells treated with ciprofloxacin exhibited a large size than the untreated ones. The co-treatment with BA resulted in a significant reduction on this effect, providing more insights into the efficacy of this compound on the suppression of the SOS pathway. Other implications of the SOS activation is the depolarization of the membrane potential and this stage is linked with the bacterial programmed cell death [54,55,56,57,58,59]. Our results indicated that BA (alone or in combination with ciprofloxacin) induces hyperpolarization in the membrane of *S. aureus*. These findings might be associated with the previous observations that BA impairs the activity of *S. aureus* electron transport chain, which results in increased levels of reactive species and cell dysfunction [49]. In addition, these results might be associated with its additive effect in the antimicrobial action of ciprofloxacin.

To further support the inhibition of the SOS response by BA, we evaluated its impacts on the mutation frequency and drug tolerance, provoked by ciprofloxacin. BA was effective in reducing the number of rifampicin- and ciprofloxacin-resistant colonies induced by the treatment with ciprofloxacin. This compound also decreased the evolution of *S. aureus* tolerance towards ciprofloxacin. These results were expected because inhibition of the SOS response is known to alter bacterial sensitivity to antibiotics [60], and this pathway is also associated with bacterial persistence and tolerance to drugs (especially those that induce DNA damage).

## 4. Materials and Methods

### 4.1. Plant Material

BA was extracted from the leaves of *Eugenia flavescens* DC [39]. The plant material was collected in the *Murieta* beach, Marine Extractive Reserve (Maracanã, Pará, Brazil). The identification was made by the botanist Luis Carlos Lobato in the herbarium of the Emilio Goeldi Paraense Museum (Belém, Brazil; voucher MG 196794). The procedure of extraction and identification of the compound was performed as described by Cantanhede Filho et al. (2018) [39].

### 4.2. Bacterial Strains

*S. aureus* ATCC 6538 was provided by the Microbial collection of *Universidade Ceuma* (São Luís, Brazil). The clinical isolate 432170 was obtained from diabetic foot ulcer of a patient with type 2 diabetes mellitus (Approved by the Ceuma University Ethics Committee N° 2517263). The susceptibility profile of *S. aureus* 432170 is shown in the Supplementary Table S1). The assays for SOS inhibition were performed using the *S. aureus* 8325-4 derivative strain, carrying a *recA::lacZ* transcriptional fusion in its chromosome [40]. 

### 4.3. Antimicrobial Activity

The antimicrobial activity was performed through the determination of MIC by the broth micro-dilution method. Briefly, serial two-fold dilutions were performed in a 96-well microplate (concentrations ranged from 5000 μg/mL to 4.88 μg/mL) in MH broth. In parallel, microbial suspensions were prepared with turbidity equivalent to a 0.5 tube of the McFarland scale (1.5 × 10^8^ CFU/mL). After this, 10 μL of each bacterial suspension were added to respective wells and the plates were incubated at 37 °C for 24 h. Then, the microbial growth was measured by the resazurin sodium oxide-reduction indicator (30 µL of 0.03% aqueous solution; Sigma-Aldrich, St. Louis, MO, USA). After 40 min of incubation, changes in color from blue to pink were classified as microbial growth [61]. The MIC was defined as the lowest concentration capable of inhibiting bacterial growth.

### 4.4. SOS Inhibition Assay

To determine the inhibitory effect of each compound against the SOS response induced by ciprofloxacin, we used a bacterial strain derived from *S. aureus* 8325-4 (*recA::lacZ*) [40]. A recent culture of this strain was diluted in MH broth (1:100) and cultivated, it until reached an optical density of 0.1 at 630 nm (OD_630_). Aliquots (500 µL) of the bacterial suspension were co-incubated with BA (100 μg/mL and 200 μg/mL) and sub-MIC of ciprofloxacin (MIC/4 = 0.0195 µg/mL). Cells incubated with vehicle (1% DMSO) were used as a negative control, while a group treated with ciprofloxacin constituted the positive control. After 3 h of incubation, the cells were disrupted with toluene (100 µL) and the levels of β-galactosidase were measured by using ONPG (2-Nitrophenyl β-d-galactopyranoside) [22]. A recent culture of this strain was diluted in MH broth (1:100) and cultivated until it reached an optical density of 0.1 at 630 nm (OD_630_). Aliquots (500 µL) of the bacterial suspension were co-incubated with BA (100 μg/mL and 200 μg/mL) and sub-inhibitory concentration (sub-MIC) of ciprofloxacin (MIC/4 = 0.0195 µg/mL). Cells incubated with the vehicle (1% DMSO) were used as a negative control, while a group treated with ciprofloxacin constituted the positive control. After 3 h of incubation, the cells were disrupted with toluene (100 µL) and the levels of β-galactosidase were measured by using ONPG (2-Nitrophenyl β-d-galactopyranoside) [22]. 

### 4.5. Effect of Betulinic Acid on the Antimicrobial Action of Ciprofloxacin

The effect of BA in the antimicrobial action of the ciprofloxacin was evaluated against *S. aureus* ATCC 6538 and a recently isolated *S. aureus* clinical strain (SA 432170). In this assay, MH broth was supplemented with BA (200 µg/mL or 100 µg/mL), which was used for serially diluted ciprofloxacin and the MIC was determined, as described above. The MIC values obtained for ciprofloxacin in the MH medium, without BA, were used as controls.

### 4.6. Evaluation of Cell Size Changes

The morphologic studies were performed using flow cytometry and fluorescence microscopy. Bacterial inoculums were made from a fresh culture of *S. aureus* ATCC 6538 (OD_630_ = 0.1), which were incubated with BA (200 µg/mL or 100 µg/mL) and ciprofloxacin (MIC/4 = 0.0195 µg/mL). After 3h, the cells were fixed and stained with acridine orange (10 µg/mL). The cell size was measured by flow cytometry (BD Accuri^TM^, BD Biosciences, San Jose, CA, USA; FSC) and fluorescence microscopy (Axio Imager Z2, Carl Zeiss, Jena, Germany).

### 4.7. Assessment of Membrane Potential Using Flow Cytometry

Changes in membrane potential following BA treatment (with or without ciprofloxacin) were estimated by labeling with Rhodamine123 dye. *S. aureus* ATCC 6538 (OD_630_ = 0.1) were incubated with BA (200 μg/mL; 100 μg/mL) and ciprofloxacin (MIC/4 = 0.0195 µg/mL) (in combination or not) for 4 h. The samples were centrifuged and pellet was washed twice with PBS (2X). Cells were resuspended into 100 μL of PBS and stained with 2.5 μL of Rhodamine123 (1 mg/mL). After 20 min of incubation (hidden of light), the cells were washed and 20,000 events were recorded by flow cytometry, using the FL1 scatter threshold (BD Accuri^TM^, USA). Changes in fluorescence intensity emission by Rho123 were measured by the variation index (VI), through the equation (MT-MC)/MC, where MC is the mean of fluorescence intensity of the control and MT is the mean of the treated cells. 

### 4.8. Determination of Mutation Frequency

Recently grown cells (OD_630_ = 0.1) were diluted (1:100) in 500 μL of MH broth and inoculated with ciprofloxacin (MIC/4 = 0.0195 µg/mL) and BA (200 μg/mL). After 12 h and 24 h of incubation, the same amount of cells were diluted into 500 μL of MH broth, supplemented with the tested compounds. After 48 h, serial dilutions were plated in MH agar or MH agar supplemented with the tested drugs (ciprofloxacin (0.125 μg/mL) or rifampicin (100 μg/mL)). The number of colony-forming units (CFU) was defined and mutation frequency was calculated by the ratio of the number of mutant cells (CIP^R^ or RIF^R^) per total CFU.

### 4.9. Assessment of Bacterial Tolerance to Ciprofloxacin

The drug-mediated tolerance assay was performed through successively cultured sub-MICs of ciprofloxacin and BA (200 μg/mL or 100 μg/mL). In each cycle, one suspension of *S. aureus* ATCC 6538 was diluted (1:50) in the MH broth containing the tested compounds. After each cycle of 24 h, the MIC for ciprofloxacin was determined. The procedure was repeated for 10 days [62].

### 4.10. Statistical Analysis 

The data were analyzed in GraphPad Prism 7.0 (GraphPad Software Inc., San Diego, CA, USA), using One-Way unidirectional variance analysis (ANOVA) and Tukey’s test. The experiments were performed in triplicates, in at least three independent assays. All results are expressed in mean values of the groups and have been analyzed by considering the value of *p* < 0.05 as statistically significant.

## 5. Conclusions

This study provided several phenotypic insights that support the inhibitory action of BA on the induction of SOS response by ciprofloxacin in *S. aureus*. This effect might be linked with the improvement of ciprofloxacin action towards *S. aureus*, and reduction on the ciprofloxacin-mediated mutagenesis and tolerance. These findings suggest that BA could be used as a lead molecule in the development of drug helpers, in an attempt to reduce the emergence of bacterial resistance. It is also important to consider the presence of the two functional groups in BA (3-OH and 17-COOH) that allow structural modifications on this molecule, which could result in the improvement of its biological activity.

## Figures and Tables

**Table 1:** Modulatory effect of betulinic acid (BA) on the Ciprofloxacin action towards *Staphylococcus aureus* strains.

*S. aureus* Strain	Ciprofloxacin	Ciprofloxacin + BA (100 µg/mL)	Ciprofloxacin + BA (200 µg/mL)
ATCC 6538	0.078 μg/mL	0.039 μg/mL	0.0195 μg/mL
432170	0.0195 μg/mL	0.097 μg/mL	0.00485 μg/mL

## Supplementary Materials

The following are available online at https://www.mdpi.com/1420-3049/24/9/1757/s1, Table S1: Susceptibility Profile of *Staphylococcus aureus* 432170 isolated from diabetic foot ulcer of a patient with type 2 diabetes mellitus.
