# Supplementary material for: Betulinic Acid Prevents the Acquisition of Ciprofloxacin-Mediated Mutagenesis in Staphylococcus aureus
Source: Molecules. 2019 May 7;24(9):1757. doi: 10.3390/molecules24091757 (PMC6539033; doi:10.3390/molecules24091757)
Supplement: Supplementary file 1 [file molecules-24-01757-s001.pdf]

Supplementary material

Supplementary Table S1: Susceptibility Profile of *Staphylococcus aureus* 432170 isolated from diabetic foot ulcer of a patient with type 2 diabetes mellitus.

	CIP	CLI/DA	DAP	ERY/E	GEN	LNZ	OXA	PEN	RIF	TMP-SMX	TEC	TCY/TE	VAN
	S	R	S	R	S	S	S	S	S	S	S	S	S
MIC (µg/mL)	≤ 0.5	≤ 0.25	0.25	≥ 8	≤ 0.5	1.00	≤ 0.25	≤ 0.03	≤ 0.5	≤ 10	≤ 0.5	≤1.00	≤ 0.5

MIC – Minimum inhibtory concentration; PEN, penicillin; OXA, oxacillin; CIP, ciprofloxacin , VAN, vancomycin; TCY/TE, tetracycline; TEC, teicoplanin; GEN, gentamicin; RIF, rifampicin; LNZ, linezolid; ERY/E, erythromycin; CLI/DA, clindamycin; DAP, Daptomycin; TMP-SMX, sulfamethoxazole trimethoprim
